# The Advanced Development in Concrete Materials

**DOI:** 10.3390/ma19102021

**Published:** 2026-05-13

**Authors:** Mustafa Batikha, Adil Tamimi, Samer Al Martini

**Affiliations:** 1 Institute of Sustainable Built Environment, School of Energy, Geoscience, Infrastructure and Society, Heriot-Watt University, Dubai P.O. Box 501745, United Arab Emirates; 2 Department of Civil Engineering, College of Engineering, American University of Sharjah, Sharjah P.O. Box 26666, United Arab Emirates; atamimi@aus.edu; 3 Department of Civil Engineering, Faculty of Engineering, Abu Dhabi University, Abu Dhabi P.O. Box 59911, United Arab Emirates; samer.almartini@adu.ac.ae

Concrete is regarded as the second most widely used material after water due to its many advantages. These include, but are not limited to, its low cost, low maintenance requirements, ease of moulding, low reliance on skilled labour, and strong resistance to fire and adverse weather conditions [1]. Therefore, there is no doubt that global concrete production reaches approximately 14 billion cubic metres, with a market value of around USD 440 billion [2]. Concrete itself may be considered a relatively sustainable material, with emissions of approximately 130 kg of CO_2_ per tonne, compared with other construction materials such as hot-rolled steel (1830 kg CO_2_ per tonne) and cold-formed steel (680 kg CO_2_ per tonne) [3]. However, its widespread use accounts for approximately 7% of the global carbon footprint [4]. This is primarily attributable to cement production, which is estimated to release more than 600 kg of CO_2_ per tonne of cement into the Earth’s atmosphere.

Numerous strategies have been proposed to decarbonise the cement industry. These include the development of novel cements, reducing the clinker-to-cement ratio, the use of alternative fuels for combustion and electricity in cement production, carbon capture and reuse, improving energy efficiency through the adoption of modern dry clinker kilns, the use of low-carbon concrete, the implementation of carbon taxation, design optimisation, and the adoption of sustainable construction methods, such as 3D Concrete Printing (3DCP) and precast concrete. Nevertheless, all of these methods present their own challenges [5].

Another concern regarding the unsustainability of concrete is its reliance on the Earth’s natural resources, as aggregates used in its production constitute approximately 75% of its composition. The global aggregate market associated with concrete production exceeds 28 billion tonnes annually [6].

A further disadvantage of concrete is the generation of construction and demolition waste (CDW) from ageing structures, which requires substantial landfill capacity. It is estimated that Europe alone produces approximately 850 million tonnes of CDW annually, accounting for 31% of global production [7].

On a more positive note, concrete has the potential to incorporate a wide range of waste materials from the environment, including glass [7,8], ceramics [1,9,10], dam dredged materials [11], rubber [12,13], plastics [14,15,16,17,18], industrial by-products [19,20,21,22,23,24,25,26], construction and demolition waste [1,7,19,25,26,27,28,29,30] and agricultural residues [31,32]. Many of these materials have been shown to enhance the properties and durability of concrete, while simultaneously helping to reduce landfill disposal. This approach not only mitigates environmental impact but also lowers construction costs, ultimately contributing to more affordable housing.

In this Special Issue, the advancement of concrete towards sustainability is explored through up-to-date research contributions from various authors. Some papers provide comprehensive reviews of specific developments in concrete, such as asphalt concrete and geopolymer concrete [33,34], while others present novel studies on the advancement of recycled aggregate concrete [35,36]. Additional contributions examine the utilisation of industrial waste in the production of ready-mix and geopolymer concretes [37,38,39,40], the development of foam concrete with enhanced resistance to salt attack [41], and the strengthening of concrete through the incorporation of steel fibres [42] and rubber waste [43].

The Guest Editors of this Special Issue express their sincere appreciation to the authors for their invaluable contributions in supporting this issue with original research. They also acknowledge the dedication, effort, and valuable time invested by the reviewers in providing high-quality evaluations of all submitted papers. Finally, the Guest Editors extend their gratitude to the publisher for supporting the publication of this important topic.
